# Supplementary material for: CO2 conversion to methane and biomass in obligate methylotrophic methanogens in marine sediments
Source: ISME J. 2019 Apr 30;13(8):2107–19. doi: 10.1038/s41396-019-0425-9 (PMC6775961; doi:10.1038/s41396-019-0425-9)
Supplement: Supplementary file 1 — Supplementary materials [file 41396_2019_425_MOESM1_ESM.docx]

**CO_2_ conversion to methane and biomass in obligate methylotrophic methanogens in marine sediments**

Xiuran Yin^1,2,3‡^, Weichao Wu^2,4‡^*, Mara Maeke^1,3^, Tim Richter-Heitmann^1^, Ajinkya C. Kulkarni^1,2,3^, Oluwatobi E. Oni^1,2^, Jenny Wendt^2,4^, Marcus Elvert^2,4^ and Michael W. Friedrich^1,2^

^1^Microbial Ecophysiology Group, Faculty of Biology/Chemistry, University of Bremen, Bremen, Germany

^2^MARUM - Center for Marine Environmental Sciences, Bremen, Germany

^3^International Max-Planck Research School for Marine Microbiology, Max Planck Institute for Marine Microbiology, Bremen, Germany

^4^Department of Geosciences, University of Bremen, Bremen, Germany

Running title: Methane formation from CO_2_ in *Methanococcoides*

Correspondence:

Michael W. Friedrich,

Microbial Ecophysiology Group, Faculty of Biology/Chemistry, University of Bremen, PO

Box 33 04 40, D-28334 Bremen, Germany

Email: michael.friedrich@uni-bremen.de

^‡^ These authors contributed equally to this work.

*Current address: Department of Biogeochemistry of Agroecosystems, University of Goettingen, Goettingen, Germany

**Clone library construction**

A clone library of archaeal 16S rRNA gene fragments (~800 bp) was constructed to confirm the accuracy of classification by using short Illumina sequences (143 base pairs). PCR was conducted with primer set of 109F/912R (Table S1) and ALLin RPH polymerase Kit (highQu, Kraichtal, Germany) according to the protocol of the manufacturer. The template cDNA was used from the heavy fractions of RNA-SIP sample of the MZ incubations amended with ^13^C-DIC and unlabeled methanol. Thermocycling was performed as follows: 95 °C for 3 min; 40 cycles at 95 °C for 30 sec, 58 °C for 45 sec and 72 °C for 45 sec; 72 °C for 10 min. Purified PCR products were ligated into the pGEM-T vector (Promega, Mannheim, Germany) and transformed into *Escherichia coli* JM109 competent cells (Promega, Mannheim, Germany) according to the manufacturer. White colonies were randomly picked and cell material directly subjected to colony PCR with the following cycling parameters: 95 °C for 5 min; 28 cycles at 95 °C for 30 sec, 55 °C for 45 sec and 72 °C for 1 min; 72 °C for 5 min. Amplicons of 8 clones were submitted to LGC Genomics (Berlin, Germany) for Sanger sequencing. Sequences have been deposited at GenBank with accession numbers from MK434328 to MK434335.

**Table S1.** Primers used in this study

| **Target gene** | **Primer** | **Reference** |
| --- | --- | --- |
| Archaeal 16S rRNA gene | 806F  (5’-ATTAGATACCCSBGTAGTCC-3’) | [1] |
| Archaeal 16S rRNA gene | 912R  (5’-GTGCTCCCCCGCCAATTCCTTTA-3’) | [2] |
| *mcr*A | ME2 mod  (5’-TCATBGCRTAGTTNGGRTAGT-3’) | [3] |
| *mcr*A | ME3’Fs  (5’-GTCNGGTGGHGTMGGSTTYAC -3’) | [4] |
| Archaeal 16S rRNA gene | Arch519F  (5’-CAGCMGCCGCGGTAA-3’) | [5] |
| Archaeal 16S rRNA gene | Arch806R  (5’-GGACTACVSGGGTATCTAAT-3’) | [6] |
| Archaeal 16S rRNA gene | 109F  (5’-ACKGCTCAGTAACACGT-3’) | [7] |

**Table.S2** Carbon recovery of methanol from incubations amended with DIC and ^13^C-methanol

| Sediment | methane, µmol | ^13^C-TIC, µmol | Ratio of  methane to CO_2_ | Carbon recovery, % |
| --- | --- | --- | --- | --- |
| SRZ | 27.0 ± 1.4 | 10.8 ± 0.7 | 2.5 ± 0.3 | 81.3 ± 1.7 |
| MZ | 28.6 ± 0.4 | 8.8 ± 0.3 | 3.2 ± 0.1 | 80.4 ± 0.4 |

^13^C-TIC (total inorganic carbon in bottle) and ^13^C-methane were quantified at harvest. Data is presented as average values (n = 3, error bar = SD). A total amount of 46.5 µmol ^13^C-methanol was amended into SIP incubation.

**Table S3.** Comparison of fold increase of archaeal 16S rRNA gene copy number in heavy fractions between “MeOH + ^13^C-DIC” and “DIC + ^13^C-MeOH” incubations

| Sample | Density (g/mL) | Archaeal 16S rRNA  copy number | Fold increase compared to ^13^C-MeOH incubation |
| --- | --- | --- | --- |
| MZ_ DIC+^13^C-MeOH | 1.823 | 9.04E+00 | - |
|  | 1.817 | 3.05E+01 | - |
|  | 1.812 | 2.82E+07 | - |
|  | 1.806 | 1.59E+03 | - |
| MZ_ MeOH+^13^C-DIC | 1.82 | 1.43E+01 | 1.59E+00 |
|  | 1.815 | 3.67E+02 | 1.21E+01 |
|  | 1.809 | 2.31E+02 | 8.20E-06 |
|  | 1.803 | 3.29E+08 | 2.07E+05 |
| SMZ_ DIC+^13^C-MeOH | 1.823 | 1.69E+02 | - |
|  | 1.817 | 3.46E+02 | - |
|  | 1.812 | 1.50E+07 | - |
|  | 1.809 | 1.32E+04 | - |
| SMZ_ MeOH+^13^C-DIC | 1.823 | 3.96E+07 | 2.35E+05 |
|  | 1.817 | 1.70E+08 | 4.90E+05 |
|  | 1.812 | 7.69E+03 | 5.11E-04 |
|  | 1.806 | 3.14E+08 | 2.37E+04 |

**Table S4.** Relative abundance of archaeal reads above 0.01%.

The gray color denotes archaea taxonomy showed in Figure 2. Pink and green colors indicate the scale of archaeal relative abundance.

**Table S5.** Methanogenesis from methanol and DIC in pure culture of *M. methylutens* grown in Widdel medium. Data is expressed as average values (n = 3).

| Substrates | δ^13^C-methane,  (‰; VPDB) | δ^13^C-DIC_day 0, (‰; VPDB) | δ^13^C-DIC_day 11, (‰; VPDB) | Methane from labeled substrate, % |
| --- | --- | --- | --- | --- |
|  |  |  |  |  |
| DIC + 5% ^13^C-MeOH | 4620 ± 160 | NA | NA | 97.1 ± 3.2 |
| MeOH + 5% ^13^C-DIC | 63.8 ± 5.5 | 4170 ± 80 | 3520 ± 82.0 | 2.3 ± 0.1 ~ 2.6 ± 0.2 |

**
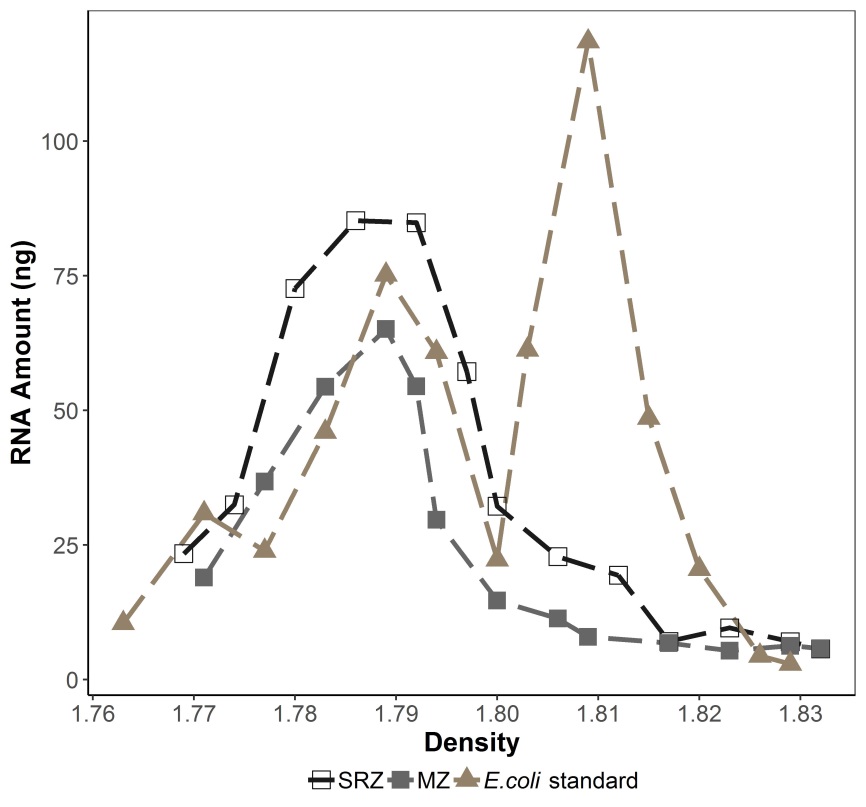
**

**Figure S1.** RNA-SIP profiles from slurry incubations amended with ^13^C-DIC only (no methanol added) and *E.coli* standard. Samples were harvested in parallel to methanol amended incubations.


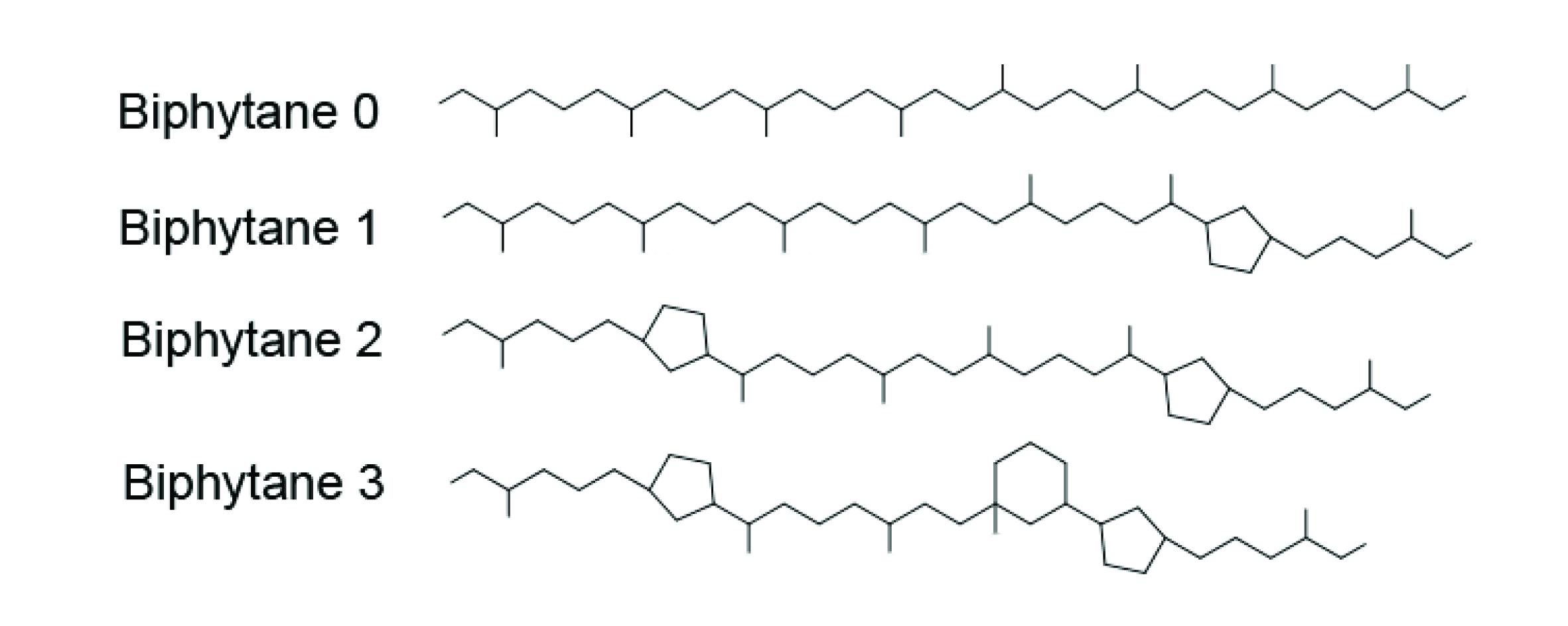


**Figure S2.** Structures of biphytane moieties released from the intact polar glycerol diphytanoyl glycerol tetraether fraction.


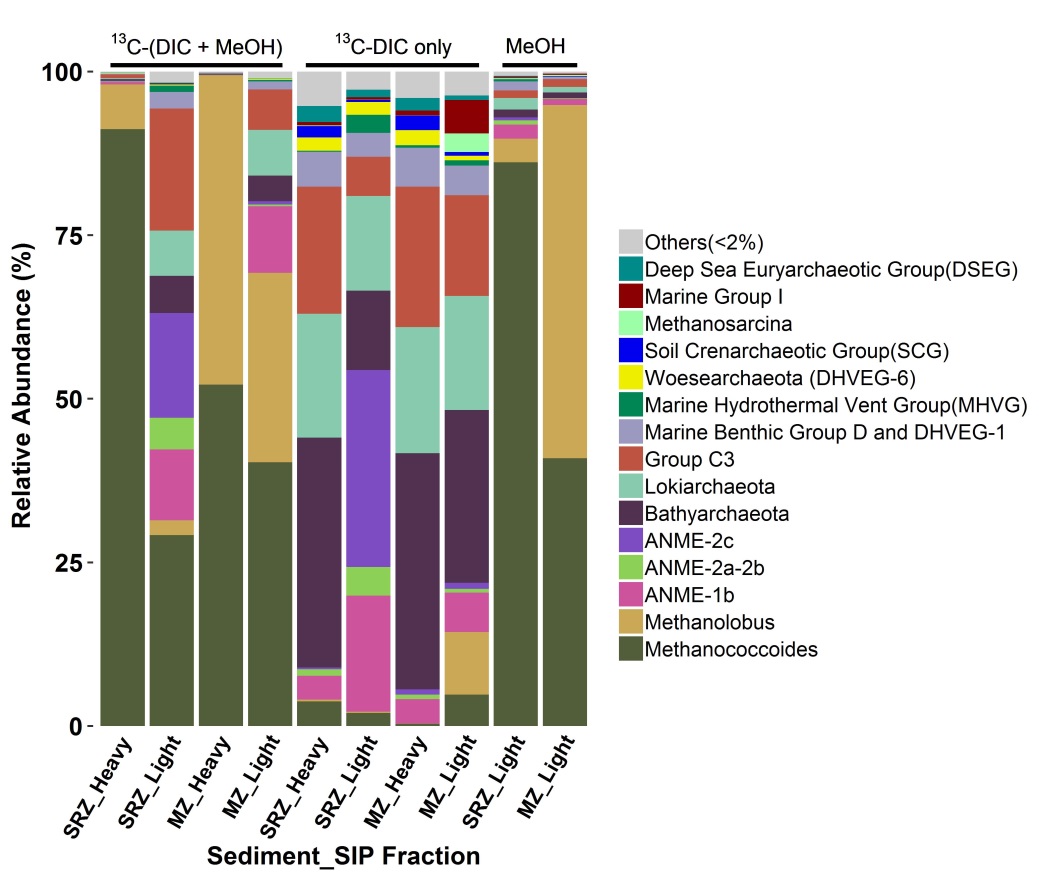


**Figure S3.** Relative abundance of archaeal 16S rRNA in the RNA-SIP samples from double-labeling incubations (^13^C-DIC + ^13^C-methanol) and control incubations (^13^C-DIC or unlabeled-methanol). No data are shown for the unlabeled-methanol controls from the SRZ and MZ due to low amount of RNA in the heavy fraction.


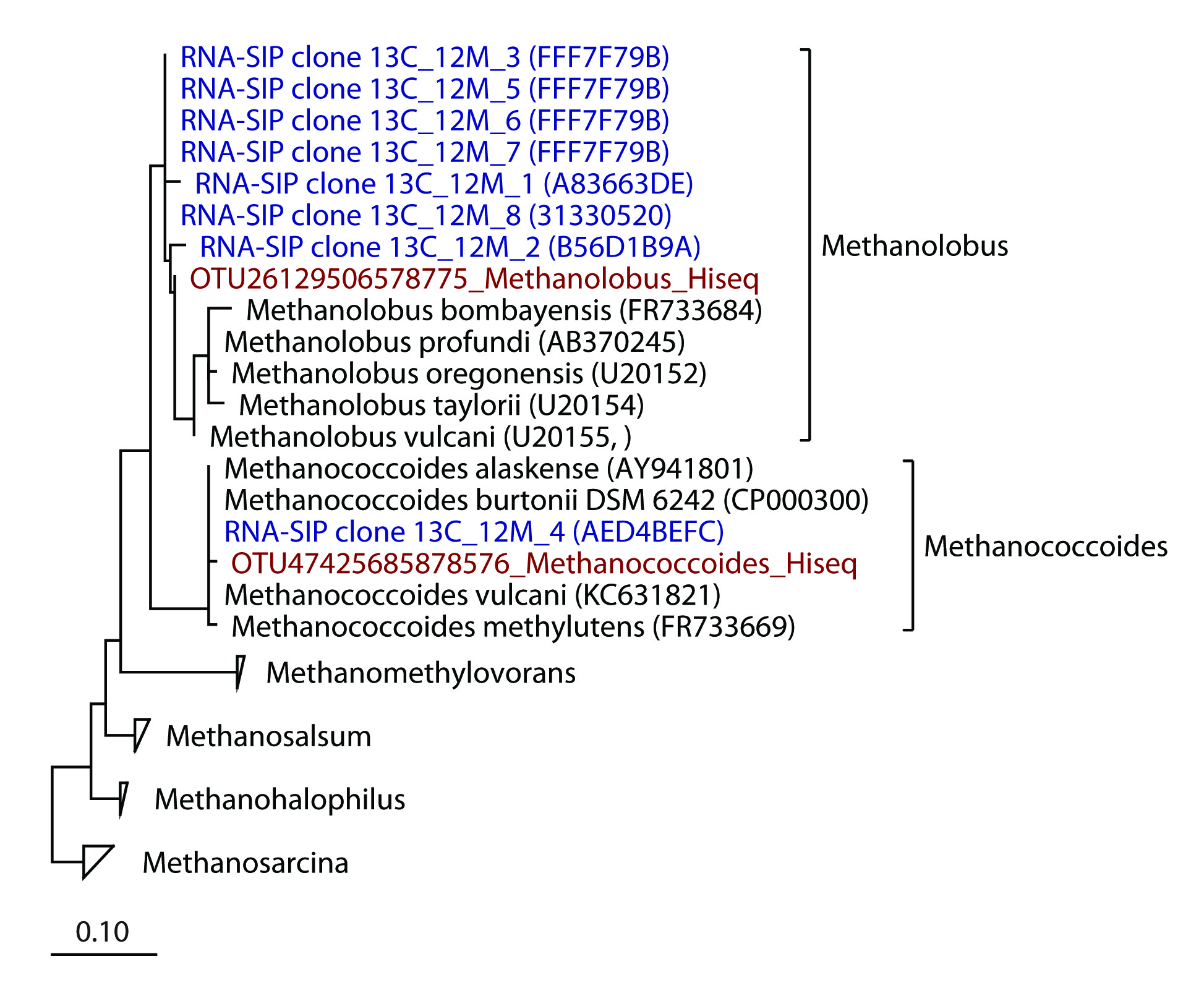


**Figure S4.** Phylogenetic tree of 16S rRNA genes from clone library (blue) and Illumina sequencing (red). Clone sequences were assembled by using SeqMan software (Version 8.0.2) and aligned online by Silva aligner (<https://www.arb-silva.de/aligner/>). The aligned sequences were input into ARB (Version 6.0.2). Aligned clone sequences and know sequences of *Methanosarcinaceae* in SILVA SSURef database (Release 132) were selected to build a phylogenetic tree using maximum likelihood algorithm and bootstrapping (n=1000). The two dominant OTUs of *Methanosarcinaceae* from Hiseq Illumina sequencing (red) were aligned and added to the tree using the ARB parismony tool.


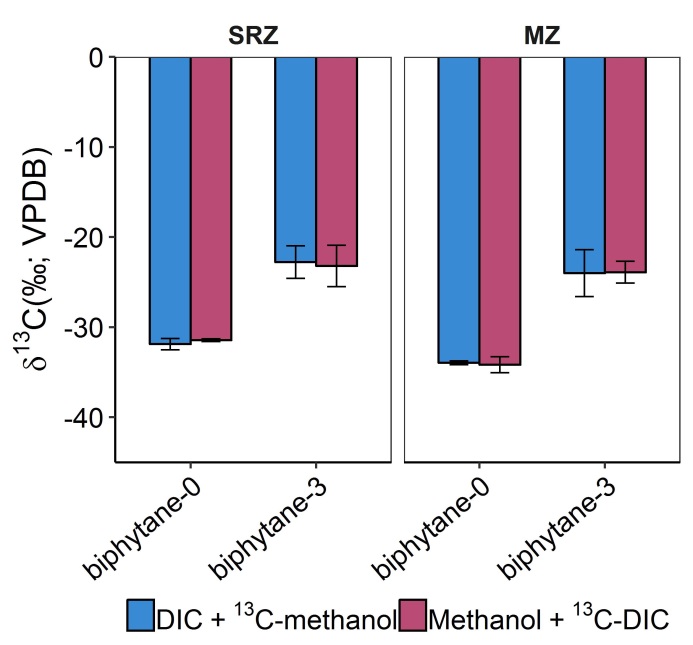


**Figure S5.** δ^13^C values of biphytanes released from the intact ploar glycerol diphytanoyl glycerol tetraether fraction. Biphytane-1 and biphytane-2 concentrations were too low to be measured accurately. Determiantion of carbon isotope values was performed after methanogenesis had ceased. Data is expressed as average values (n = 3, error bar = SD).


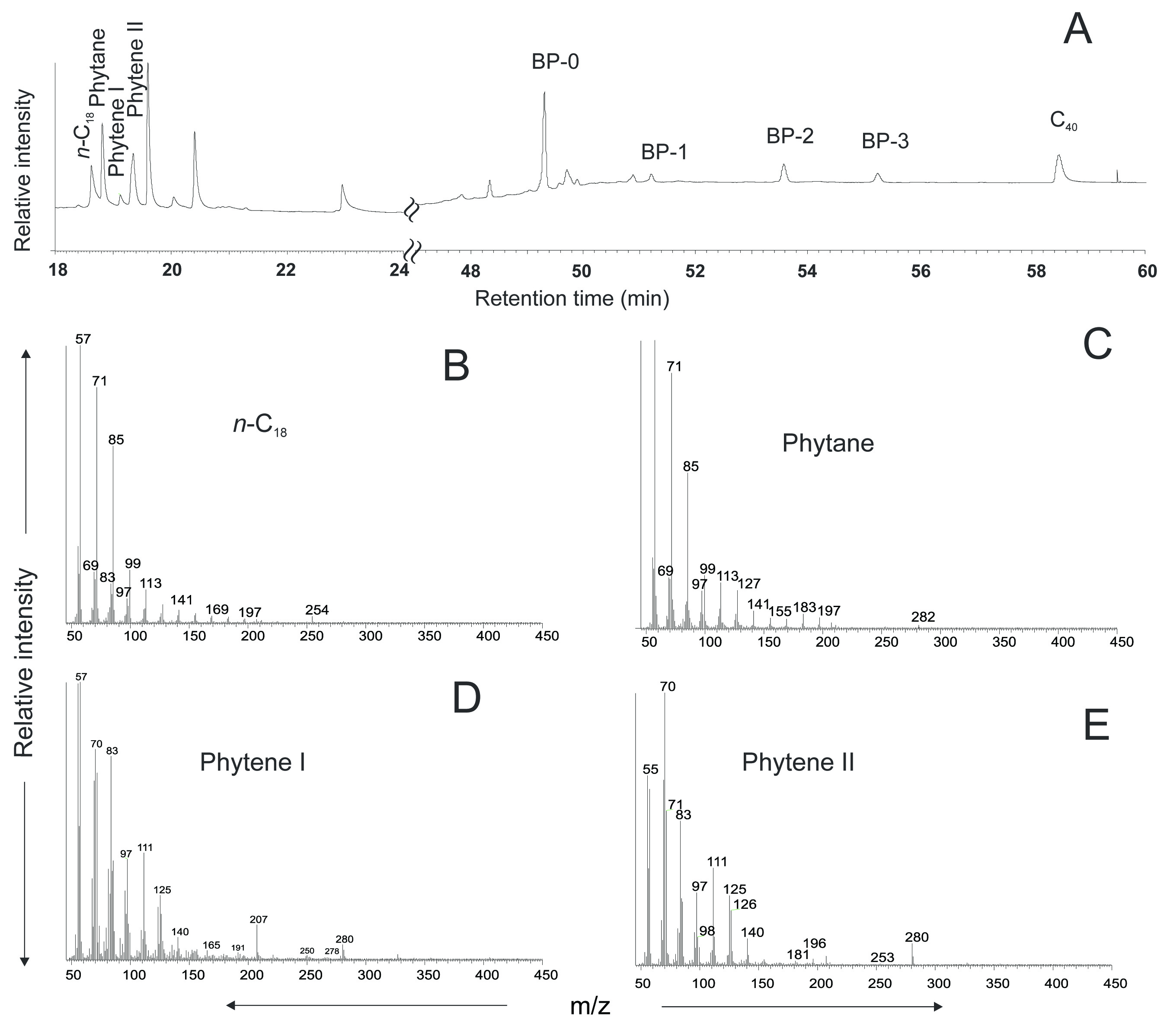


**Figure S6.** Chromatogram of phytane and phytenes released from intact polar lipid fraction in the incubations of the MZ sample amended with ^13^C-DIC and unlabeld methanol (A) and the mass spectra of compunds interests (B to E).

**Reference**

1. Yu Y, Lee C, Kim J, Hwang S. Group-specific primer and probe sets to detect methanogenic communities using quantitative real-time polymerase chain reaction. Biotechnol Bioeng. 2005;89:670-9.

2. Lueders T, Friedrich MW. Effects of amendment with ferrihydrite and gypsum on the structure and activity of methanogenic populations in rice field soil. Appl Environ Microbiol. 2002;68:2484-94.

3. Mori K, Iino T, Suzuki K, Yamaguchi K, Kamagata Y. Aceticlastic and NaCl-requiring methanogen "*Methanosaeta pelagica*" sp. nov., isolated from marine tidal flat sediment. Appl Environ Microbiol. 2012;78:3416-23.

4. Miyazaki J, Higa R, Toki T, Ashi J, Tsunogai U, Nunoura T, et al. Molecular characterization of potential nitrogen fixation by anaerobic methane-oxidizing archaea in the methane seep sediments at the number 8 Kumano Knoll in the Kumano Basin, offshore of Japan. Appl Environ Microbiol. 2009;75:7153-62.

5. Ovreas L, Forney L, Daae FL, Torsvik V. Distribution of bacterioplankton in meromictic Lake sælenvannet, as determined by denaturing gradient gel electrophoresis of PCR-ampliﬁed gene fragments coding for 16S rRNA. Appl Environ Microbiol. 1997;63:3367-73.

6. Takai K, Horikoshi K. Rapid detection and quantification of members of the archaeal community by quantitative PCR using fluorogenic probes. Appl Environ Microbiol. 2000;66.

7. Grosskopf R, Janssen PH, Liesack W. Diversity and structure of the methanogenic community in anoxic rice paddy soil microcosms as examined by cultivation and direct 16S rRNA gene sequence retrieval. Appl Environ Microbiol. 1998;64: 960-9.
